# Pycnodysostosis presented with tibial shaft fracture

**DOI:** 10.11604/pamj.2020.35.5.8581

**Published:** 2020-01-09

**Authors:** Abdelhalim Mahmoudi, Youssef Bouabdallah

**Affiliations:** 1 Department of Pediatric Surgery, University Hospital Hassan II, Fez, Morocco

**Keywords:** Pycnodysostosis, short stature, tibial shaft fracture

## Image in medicine

Pycnodysostosis is rare autosomal recessive lysosomal storage disorder of bone in which the enzyme Cathepsin K is mutated which maps on chromosome 1q21 causing osteoclast dysfunction. It is characterized by short stature, craniofacial dysmorphias, osteosclerosis, and brittle bones. Recognition of these signs is important in order to make the diagnosis and prevent possible complications. A 13-year old male patient of 130 cm height presented to the emergency with pathological fracture in shaft of left tibia. On the basis of clinical and radiological findings, he was diagnosed as a case of pycnodysostosis. The patient had history of pathological fracture in shaft of right tibia. With normal bone healing, open fontanelles, short stature, short finger tips, flat and grooved nails, micrognathia, and irregular teeth with hypodontia. Radiological evaluation of patient revealed generalized osteopetrosis, short distal phalanges, obtuse angle of mandible and open fontanelles. He was managed conservatively and showed normal bone healing pattern. No specific treatment options exist, so treatment is supportive, with fracture prevention and management constituting the most important aspects of clinical care. Dental hygiene and regular checkups are also helpful in preventing complications.

**Figure 1:**
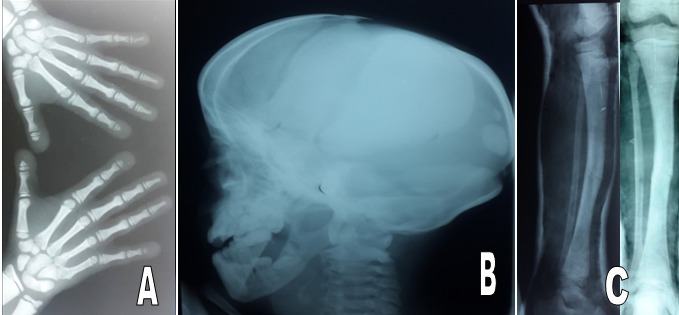
A) AP X-ray of both hands demonstrate short stubby fingers with dysplastic nails and acro-osteolysis; B) lateral skull X-rays reveal a large cranial vault with persistent open sutures and fontanelles as well as wormian bones: hypoplastic facial bones and an underdeveloped mandible with an obtuse jaw angle are also demonstrated; C) AP and lateral X-ray reveal a tibial shaft fracture

